# Intestinal epithelial BLT1 promotes mucosal repair

**DOI:** 10.1172/jci.insight.162392

**Published:** 2022-12-08

**Authors:** Shusaku Hayashi, Chithra K. Muraleedharan, Makito Oku, Sunil Tomar, Simon P. Hogan, Miguel Quiros, Charles A. Parkos, Asma Nusrat

**Affiliations:** 1 Department of Pathology, University of Michigan, Ann Arbor, Michigan, USA.; 2 Institute of Natural Medicine, University of Toyama, Toyama, Japan.

**Keywords:** Gastroenterology, Inflammation, Inflammatory bowel disease

## Abstract

Acute and chronic intestinal inflammation is associated with epithelial damage, resulting in mucosal wounds in the forms of erosions and ulcers in the intestinal tract. Intestinal epithelial cells (IECs) and immune cells in the wound milieu secrete cytokines and lipid mediators to influence repair. Leukotriene B_4_ (LTB_4_), a lipid chemokine, binds to its receptor BLT1 and promotes migration of immune cells to sites of active inflammation; however, a role for intestinal epithelial BLT1 during mucosal wound repair is not known. Here we report that BLT1 was expressed in IECs both in vitro and in vivo, where it functioned as a receptor not only for LTB_4_ but also for another ligand, resolvin E1. Intestinal epithelial BLT1 expression was increased when epithelial cells were exposed to an inflammatory microenvironment. Using human and murine primary colonic epithelial cells, we reveal that the LTB_4_/BLT1 pathway promoted epithelial migration and proliferation leading to accelerated epithelial wound repair. Furthermore, in vivo intestinal wound repair experiments in BLT1-deficient mice and bone marrow chimeras demonstrated an important contribution of epithelial BLT1 during colonic mucosal wound repair. Taken together, our findings show a potentially novel prorepair in IEC mechanism mediated by BLT1 signaling.

## Graphical abstract

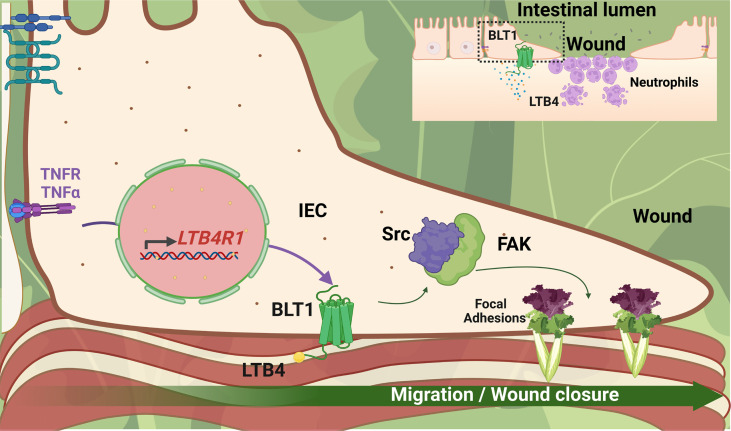

## Introduction

The gastrointestinal epithelium serves as a highly regulated protective barrier against luminal antigens and microbes. Acute and chronic intestinal inflammation is associated with epithelial damage, resulting in mucosal wounds in the form of erosions and ulcers. In response to injury, intestinal epithelial cells (IECs) have a remarkable capacity to migrate and proliferate to cover denuded surfaces and restore the critical epithelial barrier. Such reparative events are orchestrated by the spatiotemporal crosstalk between epithelial cells and infiltrating and resident immune cells including neutrophils, monocytes, macrophages, as well as stromal cells (1). Epithelial and immune cells in the wound milieu secrete mediators including cytokines and specialized proresolvin lipid mediators (SPMs) to influence repair. Many SPMs bind to G protein–coupled receptors (GPCRs) and promote resolution of inflammation (2, 3). Recently we reported that the SPM resolvin E1 (RvE1) promotes intestinal epithelial wound repair by increasing migration and proliferation of IECs (4). Receptors for RvE1 include BLT1, a high-affinity receptor for leukotriene B_4_ (LTB_4_) and ChemR23, also known as CMKLR1 (5). While BLT1 expression and function in immune cells such as neutrophils has been extensively studied (6–12), a few reports of epithelial BLT1 in the lungs are published; little is known about IEC expression of BLT1 and associated receptor-mediated signaling events (13, 14).

BLT1 agonists LTB_4_ and RvE1 trigger distinct responses when binding BLT1 in immune cells. While LTB_4_ serves as a chemotactic signal critical in regulation of immune cell migration to sites of active inflammation (15), RvE1 is described as an agonist that binds to the receptor but does not trigger downstream signaling. Enhanced activation of the LTB_4_/BLT1 pathway occurs in conditions associated with pathologic intestinal inflammation as observed in inflammatory bowel disease (IBD) and colonic adenocarcinoma (16–18). LTB_4_ is increased in the colonic mucosa (16) and supernatant of ex vivo cultured colorectal biopsy specimens from individuals with IBD (17) and in serum from patients with colorectal cancer (18). These findings suggest that the LTB_4_/BLT1 pathway plays an important role in the pathophysiology of a diverse set of intestinal diseases.

In the present study, we investigated the role of BLT1 in regulation of colonic epithelial wound repair. We demonstrate by in vitro and in vivo approaches that IECs express BLT1 in a temporal manner, with increased expression after exposure to proinflammatory conditions. Using primary cultures of human and murine colonic epithelial cells (colonoids), we show that LTB_4_ ligation of BLT1 promotes epithelial migration and proliferation leading to increased wound repair. Furthermore, we show that wound repair is delayed in BLT1-KO (B6.129Sa-*Ltb4r1tm1Adl*/J; *Ltb4r1^–/–^*) mice, and bone marrow (BM) transplant experiments demonstrate critical contributions of nonhematopoietic BLT1-expressing cells in colonic mucosal wound repair. We observe that prorepair effects of the LTB_4_/BLT1 axis align with signaling events that regulate cell matrix focal adhesions and cell migration. Collectively, these data identify an important prorepair function of epithelial BLT1 signaling in promoting intestinal mucosal wound healing.

## Results

### BLT1 functions as a major epithelial receptor for RvE1.

**Figure 1:**
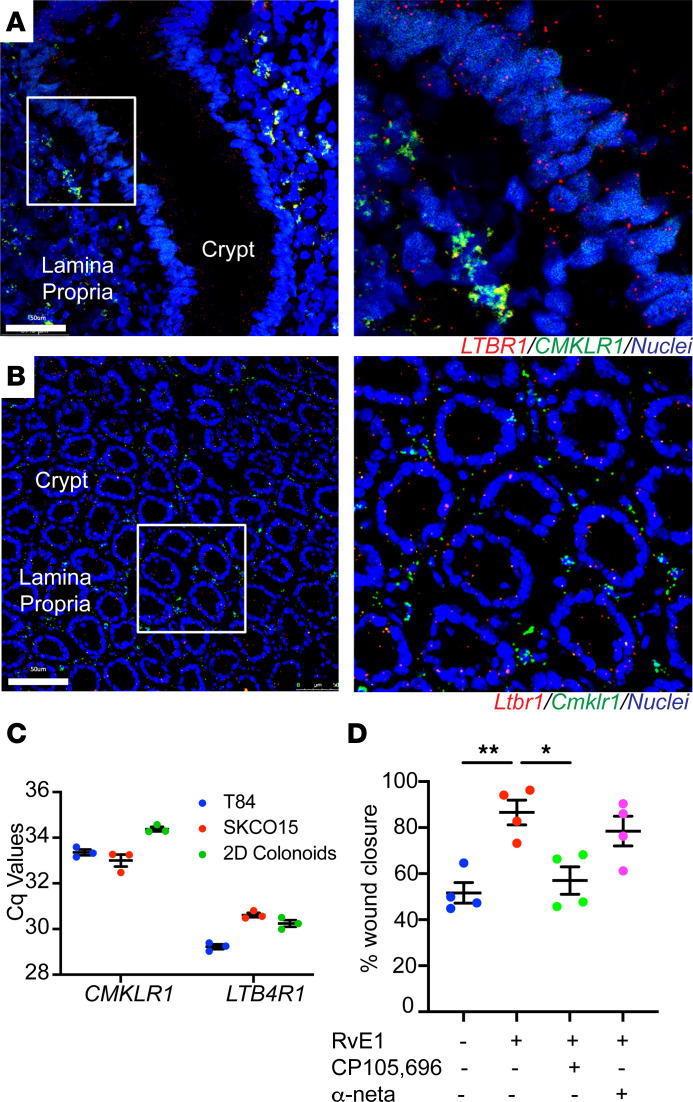
BLT1 functions as a major epithelial receptor for RvE1. (**A**) RNAscope staining for *LTB4R* and *CMKLR1* mRNA expression in frozen sections from colonic tissue of humans. (**B**) RNAscope staining for *Ltb4r1* and *Cmklr1* mRNA expression in frozen sections from colonic tissue of mice. Scale bars: 50 μm. (**C**) qPCR analysis of the expression of *CMKLR1* and *LTB4R* mRNA in the SKCO-15, T84, and human 2D colonoids. The data are presented as the mean ± SEM. Cq, quantification cycle (measured as cycles). (**D**) Effect of BLT1 antagonist on the prorepair activity of RvE1 in the scratch wound assay using human primary IECs. After scratch wound was produced, IECs were incubated with RvE1 (100 nM) for 24 hours. BLT1 (CP105,696; 1 μM) or CMKLR1 (α-NETA; 10 μM) antagonist was applied 30 minutes before RvE1 treatment. Quantification of wound repair at 24 hours after wounding is shown. The data are presented as the mean ± SEM. Statistical analysis was performed using 1-way ANOVA followed by post hoc Welch’s *t* test with Bonferroni’s correction. *P < 0.05, **P < 0.01, compared with RvE1.

We previously reported that RvE1 functions as a potent prorepair molecule that promotes intestinal epithelial wound healing (4). To further investigate how RvE1 activates signaling in epithelial cells to facilitate repair, we examined expression of the 2 known RvE1 receptors: BLT1 and CMKLR1. Given the lack of specific BLT1 and CMKLR1 antibodies, we analyzed spatial expression of these receptors in the human and murine colonic mucosa by RNAscope in situ hybridization. While *LTB4R/Ltb4r1* (BLT1 gene name) mRNA was expressed in the colonic epithelium and lamina propria, *CMKLR1/Cmklr1* mRNA was detected *only* in lamina propria cells (Figure 1, A and B). Given that BLT1 has been reported to be predominantly expressed by immune cells and CMKLR1 expression has been reported in immune and epithelial cells, this was an unexpected finding. To corroborate these results, we performed quantitative PCR (qPCR) on human intestinal epithelial cell lines and primary epithelial cultures (SKCO-15, T84, and colonoids grown as monolayers). Such analyses revealed that IECs expressed 16-fold more *LTB4R* than *CMKLR1*, indicating that CMKLR1 expression is low in IECs (Figure 1C). To determine if BLT1 contributes to the prorepair activity of RvE1 in IECs, we examined the effect of a BLT1 antagonist on RvE1-induced epithelial wound healing in vitro using primary human colonic epithelial cells (colonoids) cultured as 2-dimensional (2D) monolayers. Time-lapse imaging of healing wounds using human colonoids demonstrated that the increased wound repair induced by RvE1 (100 nM) was inhibited by incubation with a selective BLT1 antagonist, CP105,696 (1 μM) (Figure 1D). A similar effect on wound repair was obtained in primary murine colonic epithelial monolayers incubated with this BLT1 antagonist (Supplemental Figure 1A; supplemental material available online with this article; https://doi.org/10.1172/jci.insight.162392DS1). Although CMKLR1 is a RvE1 receptor, pretreatment with the selective CMKLR1 antagonist α-NETA (10 μM) did not abolish the prorepair response triggered by RvE1 in human and murine primary IECs (Figure 1D and Supplemental Figure 1A). To further determine the interaction of RvE1 and BLT1, we performed a computational docking simulation, which is a useful technique to calculate intra- and intermolecular energies of target receptors and ligands. Results of the analysis supported binding of RvE1 to BLT1 (Supplemental Figure 1B). Collectively, our findings suggest that BLT1 is expressed in the intestinal epithelia and functions as a receptor for RvE1 during wound repair.

### Epithelial BLT1 is upregulated in response to colonic mucosal injury.

**Figure 2:**
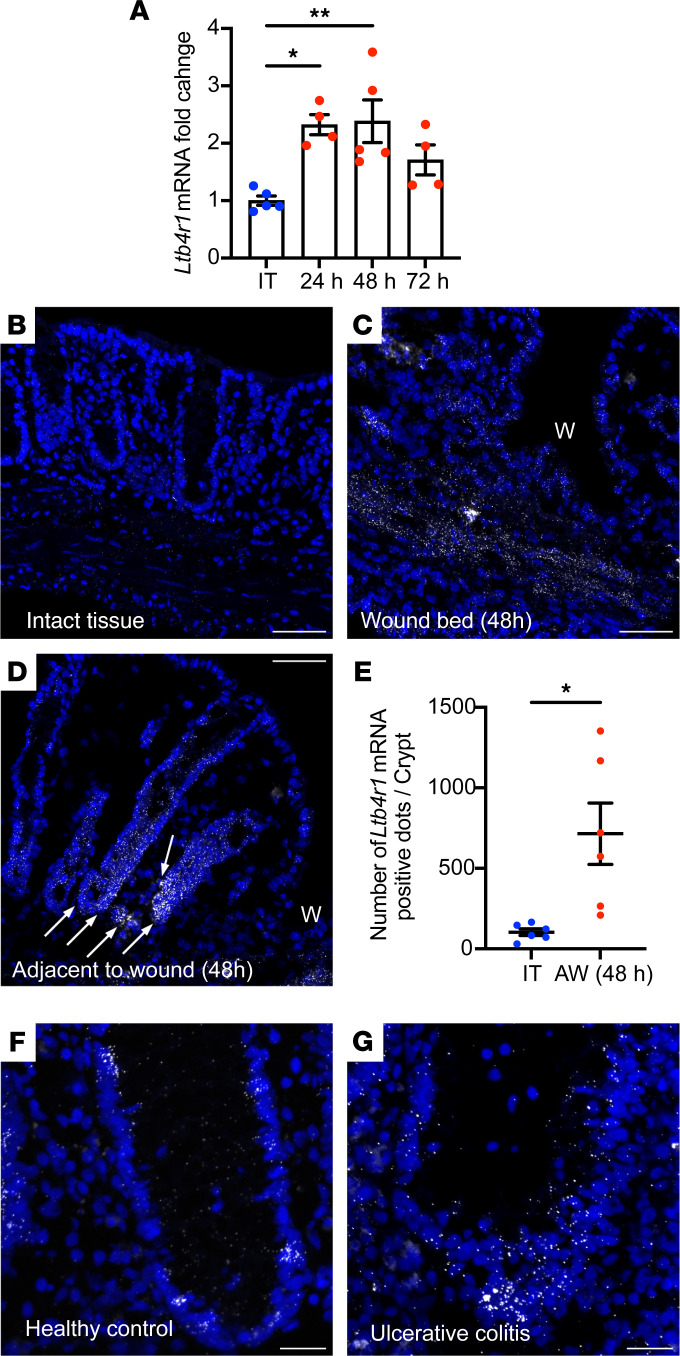
Epithelial BLT1 is upregulated in response to colonic mucosal injury. (**A**) The changes in the expression of *Ltb4r1* mRNA in 3 mm punch biopsies of intact colonic tissues and colonic mucosal wounds on different days after injury. The data are presented as the mean ± SEM of 4–5 mice. Statistical analysis was performed using 1-way ANOVA followed by post hoc Welch’s *t* test with Bonferroni’s correction. *P < 0.05, **P < 0.01, compared with intact tissue (IT). (**B**–**D**) RNAscope staining for *Blt1* mRNA in frozen sections from intact tissues and wounded colonic tissues 2 days after injury. Arrows indicate upregulation of Ltb4r1 expression in the crypts next to the wound. W, wound. Scale bar is 50 μm. (**E**) The number of *Ltb4r1* mRNA–positive dots in the crypt of intact colonic tissues and colonic mucosal wounds (adjacent to wound) 2 days after injury is shown. The data are presented as the mean ± SEM of 6 mice. Statistical analysis was performed using an unpaired (2-tailed) *t* test with Welch’s correction. *P < 0.05, compared with IT. AW, adjacent to wound. (**F** and **G**) RNAscope staining for *LTB4R* mRNA expression in frozen sections from healthy controls and patients with ulcerative colitis.

To investigate the role of BLT1 in intestinal mucosal wound repair, *Ltb4r1* mRNA expression and spatial localization were analyzed in healing biopsy-induced murine colonic mucosal wounds. qPCR analyses of harvested mucosal colonic wounds revealed that *Ltb4r1* mRNA was significantly upregulated 24 and 48 hours after injury (Figure 2A). Additionally, *Ltb4r1* mRNA was detected in the colonic epithelium and in lamina propria cells of murine mucosa by RNAscope in situ hybridization (Figure 2B). *Ltb4r1* mRNA expression was increased in the wound bed and epithelium adjacent to wounds 48 hours after injury (Figure 2C). We observed *Ltb4r1* mRNA was highly expressed in IECs located at the bases of crypts adjacent to healing wounds (Figure 2D). Quantification of these findings revealed *Ltb4r1* mRNA was upregulated 6.9-fold 48 hours after injury (Figure 2E). Since many mucosal inflammatory diseases such as IBD are associated with mucosal wounds, we examined expression of *LTB4R* mRNA in tissue sections from samples from individuals with IBD (active ulcerative colitis). Importantly, epithelial *LTB4R* mRNA was increased in colonic crypts from IBD biopsy samples (Figure 2G) compared with uninflamed controls (Figure 2F). These results are consistent with the concept of upregulated expression of intestinal epithelial BLT1 in response to mucosal inflammation and injury in vivo.

Our findings suggested that BLT1 is preferably expressed in the base of human and murine colonic crypts, colocalizing with stem cell markers, Lgr5 and HopX (Supplemental Figure 2). At the crypt base, proliferative crypt epithelial cells differentiate and migrate toward the luminal surface. To further examine BLT1 expression in proliferative crypt base colonic epithelial cells versus differentiated luminal epithelial cells, we examined *LTB4R*/*Ltb4r1* mRNA expression in primary IEC cultures and colonoids differentiated in vitro. Colonoids in Wnt-containing media cultured as 3D cysts are known to contain stem-like/proliferative epithelial cells. Such 3D structures can be dissociated and cultured as differentiated 2D monolayers. As shown in Supplemental Figure 2, *LTB4R/Ltb4r1* mRNA expression was significantly higher in 3D cultured colonoids compared with 2D differentiated monolayers in both human and murine colonic epithelial cells. These results suggest preferential expression of BLT1 in proliferative colonic crypt base epithelial cells that is upregulated in response to injury. Unfortunately, these results could not be correlated with protein expression as specific commercial BLT1 antibodies required for these analyses are not available (Supplemental Figure 3A). We did observe significantly higher levels of LTB_4_ in healing colonic wounds compared with intact healthy tissue, indicating that an increase in the receptor mRNA expression correlates with higher ligand secretion in vivo (Supplemental Figure 3B).

### BLT1 regulates intestinal epithelial wound repair.

**Figure 3:**
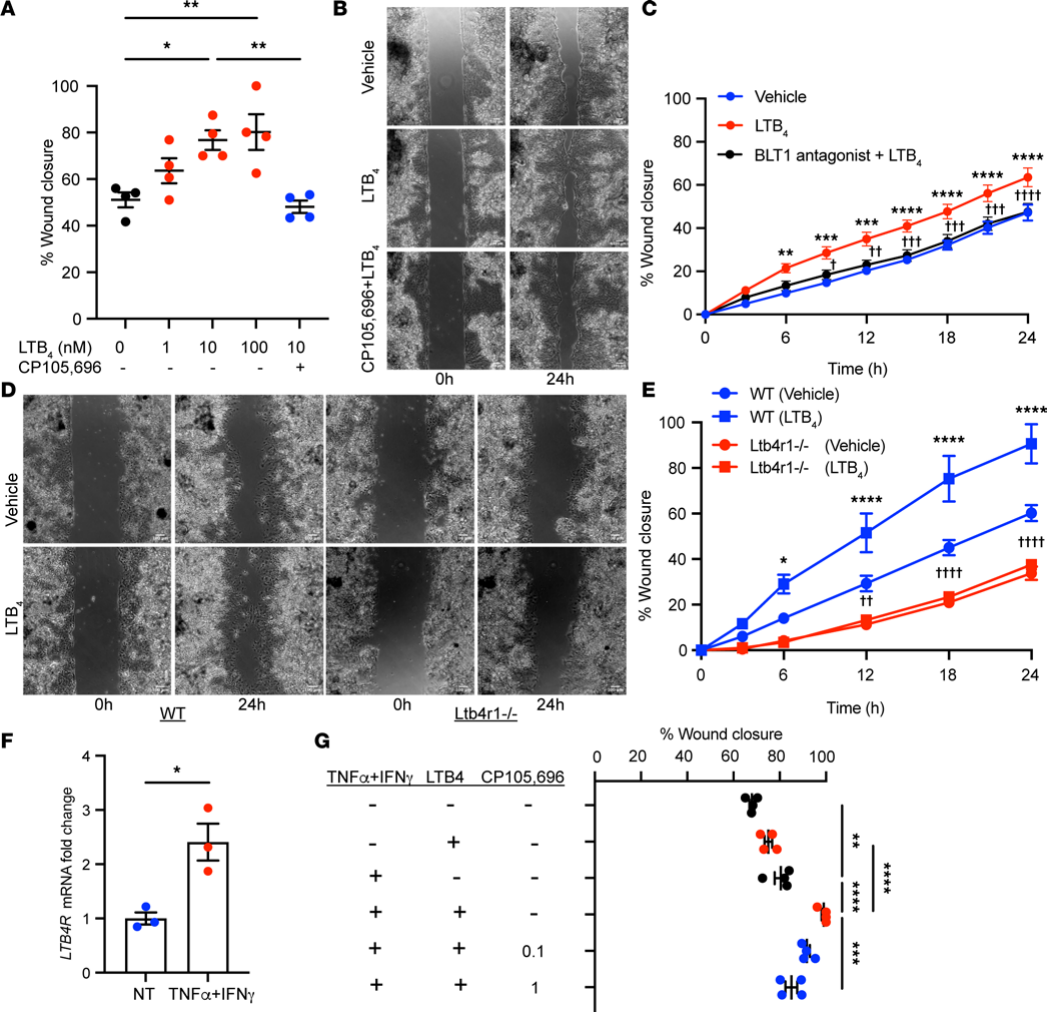
BLT1 regulates intestinal epithelial wound repair. (**A**) Effect of LTB_4_ in the scratch wound assay using SKCO-15 cells. The data are presented as the mean ± SEM. Statistical analysis was performed using 1-way ANOVA followed by post hoc Welch’s *t* test with Bonferroni’s correction. *P < 0.05; **P < 0.01. (**B** and **C**) Effect of LTB_4_ in the scratch wound assay using human primary colonic epithelial monolayers. (**B**) Representative phase-contrast images at 0 and 24 hours after wounding are shown. Scale bar is 100 μm. (**C**) Quantification of change over time in wound repair is shown. The data are presented as the mean ± SEM. Statistical analysis was performed using 2-way ANOVA followed by post hoc Welch’s *t* test with Bonferroni’s correction. **P < 0.01; ***P < 0.001; ****P < 0.0001, compared with vehicle. ^†^*P* < 0.05; ^††^*P* < 0.01: ^†††^*P* < 0.001; ^††††^*P* < 0.0001, compared with LTB_4_. (**D** and **E**) Effect of LTB_4_ in the scratch wound assay using primary epithelial monolayers. (**D**) Representative phase-contrast images at 0 and 24 hours after wounding are shown. Scale bar is 100 μm. (**E**) Quantification of change over time in wound repair is shown. The data are presented as the mean ± SEM. Statistical analysis was performed using 2-way ANOVA followed by post hoc Welch’s *t* test with Bonferroni’s correction. *P < 0.05; ****P < 0.0001, compared with WT (vehicle). ^††^*P* < 0.01; ^††††^*P* < 0.0001, compared with *Ltb4r1^–/–^* (vehicle). (**F**) qPCR analysis of the changes in the expression of *LTB4R* mRNA in the human 2D cultured colonoid stimulated with IFN-γ (10 ng/mL) and TNF-α (10 ng/mL). The data are presented as the mean ± SEM. Statistical analysis was performed using an unpaired (2-tailed) *t* test with Welch’s correction. *P < 0.05. NT, nontreated. (**G**) Effect of IFN-γ (100 ng/mL) and TNF-α (100 ng/mL) on the prorepair activity of low-dose LTB_4_ (1 nM) in the scratch wound assay using SKCO-15 cells. The data are presented as the mean ± SEM. Statistical analysis was performed using 1-way ANOVA followed by post hoc Welch’s *t* test with Bonferroni’s correction. **P < 0.01; ***P < 0.001; ****P < 0.0001.

To determine the role of the LTB_4_/BLT1 axis in regulating epithelial wound repair, we evaluated the effect of LTB_4_ using a well-studied and stable agonist of BLT1 on epithelial repair in model human IECs (SKCO-15). As shown in Figure 3A, LTB_4_ (1–100 nM) enhanced IEC wound repair in a concentration-dependent manner. Pretreatment with the selective BLT1 antagonist CP105,696 (1 μM) abolished the prorepair response elicited by LTB_4_ (10 nM) (Figure 3A). Furthermore, the response was replicated in healing scratch-wounded primary human colonoid cultures (Figure 3, B and C). To further verify that epithelial BLT1 activation promotes IEC wound healing, we generated primary colonic epithelial monolayers from colonoids of WT and BLT1-deficient (*Ltb4r1^–/–^*) mice. Time-lapse imaging of healing wounds in these cells revealed that LTB_4_ (10 nM) significantly enhanced wound repair in the IECs from WT mice and was not observed in colonoids derived from *Ltb4r1^–/–^* mice over a period of 24 hours (Figure 3, D and E). Importantly, wound closure was significantly delayed in primary epithelial cells lacking BLT1 compared with WT control (Figure 3, D and E).

It is now appreciated that an inflammatory milieu in wounded mucosa modulates epithelial reparative responses. We have previously reported that the cytokine tumor necrosis factor-α (TNF-α) is elevated within inflamed intestinal mucosa, where it contributes to wound repair by increasing expression of GPCRs such as PAFR (19). Furthermore, the proinflammatory cytokine interferon-γ (IFN-γ) has been observed to upregulate TNF-α receptor expression in IECs. We investigated if TNF-α and IFN-γ modulate BLT1 expression in IECs. The expression of *LTB4R* mRNA was synergistically increased by combined incubation of IECs with IFN-γ and TNF-α in primary human 2D colonoids (Figure 3F) and 3D colonoids (Supplemental Figure 4). In parallel, we examined the influence of combined stimulation with IFN-γ and TNF-α on the prorepair effect of LTB_4_ on IEC monolayers that were scratch wounded. As previously published, stimulation with IFN-γ and TNF-α (100 ng/mL each) significantly promoted wound closure in SKCO-15 model IECs (Figure 3G) (19). SKCO-15 cells pretreated with IFN-γ and TNF-α (100 ng/mL each) and then incubated with low-dose LTB_4_ (1 nM) further increased IEC wound repair when compared with LTB_4_ incubation without cytokine pretreatment (Figure 3G). We confirmed specificity of the LTB_4_ increase in IEC wound healing by treating SKCO-15 cells with the BLT1 antagonist CP105,696 (0.1–1 μM). Importantly, CP105,696 significantly inhibited the IEC enhanced wound healing promoted by LTB_4_ in combination with IFN-γ/TNF-α in a concentration-dependent manner (Figure 3G). These results suggest that an inflammatory microenvironment in the intestinal mucosa upregulates intestinal epithelial BLT1 expression, which potently promotes wound repair.

### BLT1 activation promotes migration and proliferation of IECs.

**Figure 4:**
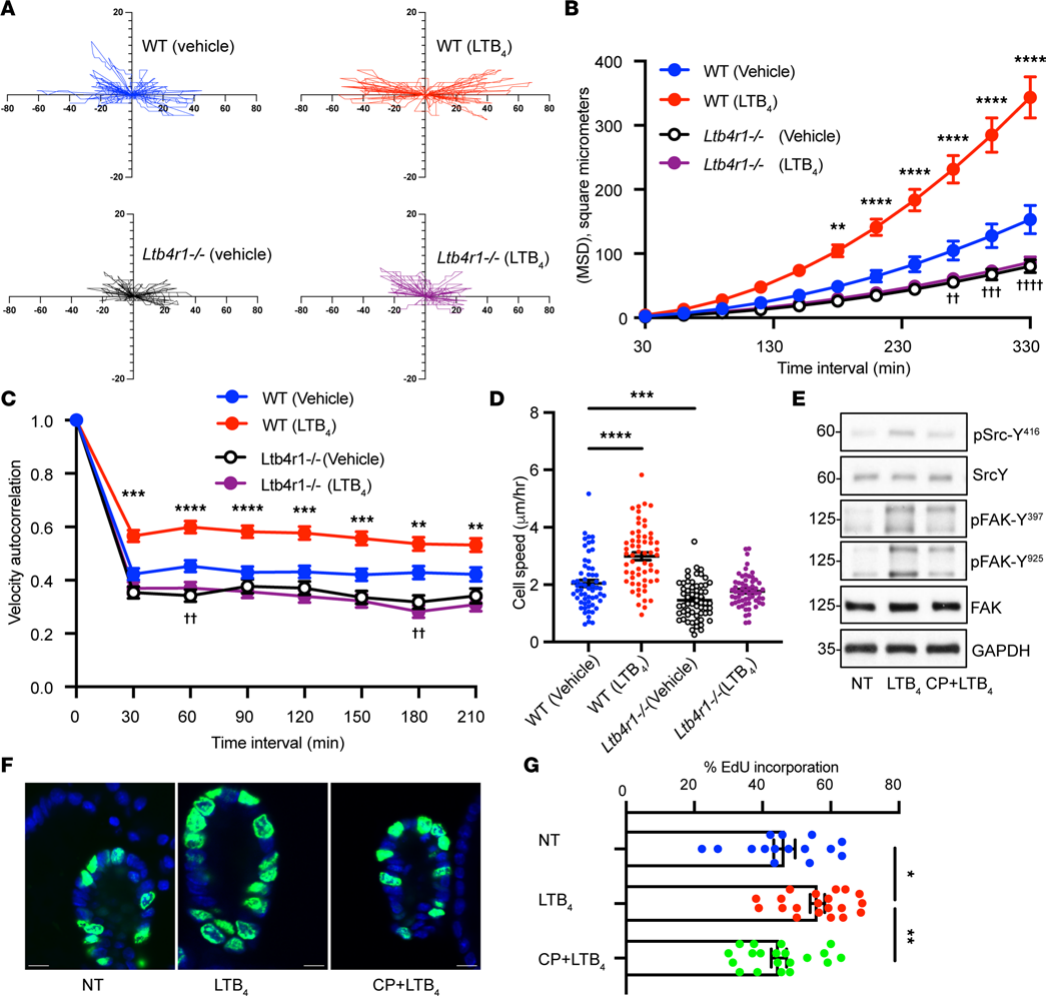
BLT1 activation promotes migration and proliferation of IECs. (**A**–**D**) Migration analysis by DiPer. (**A**) Plot at the origin graph of 20 cells. (**B**) Mean square displacement of 20 cells. The data are presented as the mean ± SEM. Statistical analysis was performed using 2-way ANOVA followed by post hoc Welch’s *t* test with Bonferroni’s correction. **P < 0.01, ****P < 0.0001, compared with WT (vehicle). ††*P* < 0.01, †††*P* < 0.001, ††††*P* < 0.0001, compared with *Ltb4r1^–/–^* (vehicle). (**C**) Velocity autocorrelation was measured on at least 20 cells. Statistical analysis was performed using 2-way ANOVA followed by post hoc Welch’s *t* test with Bonferroni’s correction. **P < 0.01, ***P < 0.001, ****P < 0.0001, compared with WT (vehicle). ††*P* < 0.01, compared with *Ltb4r1^–/–^* (vehicle). (**D**) Average cell speed was calculated on 20 cells. The data are presented as the mean ± SEM. Statistical analysis was performed using 1-way ANOVA followed by post hoc Welch’s *t* test with Bonferroni’s correction. ***P < 0.001, ****P < 0.0001. (**E**) Immunoblotting was performed on lysates from scratch-wounded IEC monolayers treated with LTB_4_ (100 nM) or vehicle. Levels of phosphorylated SRC (p-SRC) (Y416) and p-FAK (Y397, Y925) were compared with total Src, FAK, and GAPDH to assess activation. Numbers on the left represent kDa. (**F** and **G**) EdU incorporation analysis in murine 3D cultured colonoids stimulated with LTB_4_ (10 nM) for 24 hours. (**F** and **G**) Effect of BLT1 antagonist. Pictures show representative images of EdU-incorporated (shown in green) colonoids. Blue, nuclei. Scale bar is 10 μm. The data are presented as the mean ± SEM. Statistical analysis was performed using 1-way ANOVA followed by post hoc Welch’s *t* test with Bonferroni’s correction. *P < 0.05, **P < 0.01.

Since it is well appreciated that collective IEC migration and proliferation orchestrate repair after injury, we investigated whether the activation of BLT1 promotes intestinal epithelial cell migration during repair by recording cell movement of wounded monolayers over 12 hours by time-lapse microscopy. Cell motility was tracked by analyzing centroid location of individual cells during the assay (Supplemental Figure 5). As shown in Figure 4A, Plot_At_Origin showed that primary cultures of LTB_4_-treated colonic epithelial cells derived from WT murine colonoids moved faster and straighter than vehicle-treated cells. Importantly, there was no difference in cell movement between LTB_4_-treated and vehicle-treated *Ltb4r1^–/–^* primary IECs. Furthermore, the movement of primary IECs derived from *Ltb4r1^–/–^* murine colonoids was slower than those of WT murine colonoids (Figure 4A). DiPer software-based analyses (20) demonstrated that mean square displacement (MSD), a classic index that provides information about directional persistence and speed, was significantly increased in murine WT IECs treated with LTB_4_ (Figure 4B). However, in *Ltb4r1^–/–^* IECs, MSD was significantly decreased compared with WT IECs (Figure 4B). Autocorrelation of cell direction, which reflects cell direction persistence by determining angles of vectors tangent to a cell’s trajectory, revealed that LTB_4_ significantly promoted cell direction persistence in WT but not *Ltb4r1^–/–^* IECs (Figure 4C). Finally, we calculated cell speed during cell migration. Treatment with LTB_4_ significantly increased cell speed in WT but not *Ltb4r1^–/–^* IECs, indicating that cell speed in *Ltb4r1^–/–^* IECs was significantly slower than that observed in WT in the presence of LTB_4_ (Figure 4D). To explore mechanisms by which the LTB_4_/BLT1 axis promotes migration of IECs, we analyzed signaling pathways that have been shown to promote epithelial migration and wound repair. Phosphorylation/activation of Src and FAK were examined using murine 2D colonoids. Grid-scratched primary IEC monolayers were incubated with LTB_4_ for 8 hours followed by analyses. We observed increased Src (Y416) and FAK (Y397 and Y925) phosphorylation in IECs treated with LTB_4_, consistent with activation of pathways playing important roles in the regulation of cell matrix turnover and forward cell movement during migration (Figure 4E). Importantly, increased phosphorylation of Src at Y416 and FAK at Y397 and Y925 was abrogated when the BLT1 antagonist CP105,696 was added in combination with LTB_4_. Since wound closure is mediated by epithelial migration and proliferation, we investigated the role of BLT1 in IEC proliferation. The effect of LTB_4_ on the incorporation of thymidine analog 5-ethynyl-2′-deoxyuridine (EdU) in murine 3D cultured colonoids was analyzed. LTB_4_ (10 nM for 24 hours) resulted in significantly increased proliferation of murine IECs (Figure 4, F and G). Importantly, the increase in LTB_4_-induced epithelial cell proliferation was significantly inhibited by pretreatment with BLT1 antagonist CP105,696 (Figure 4, F and G). To verify specificity of BLT1 in enhancing proliferation of colonic epithelial cells, we examined the effect of LTB_4_ on colonoids derived from *Ltb4r1^–/–^* mice. Indeed, stimulation with exogenously added LTB_4_ did not significantly alter proliferation of colonoids derived from mice lacking the BLT1 receptor (Supplemental Figure 6).

### Role of BLT1 in intestinal mucosal wound repair in vivo.

**Figure 5:**
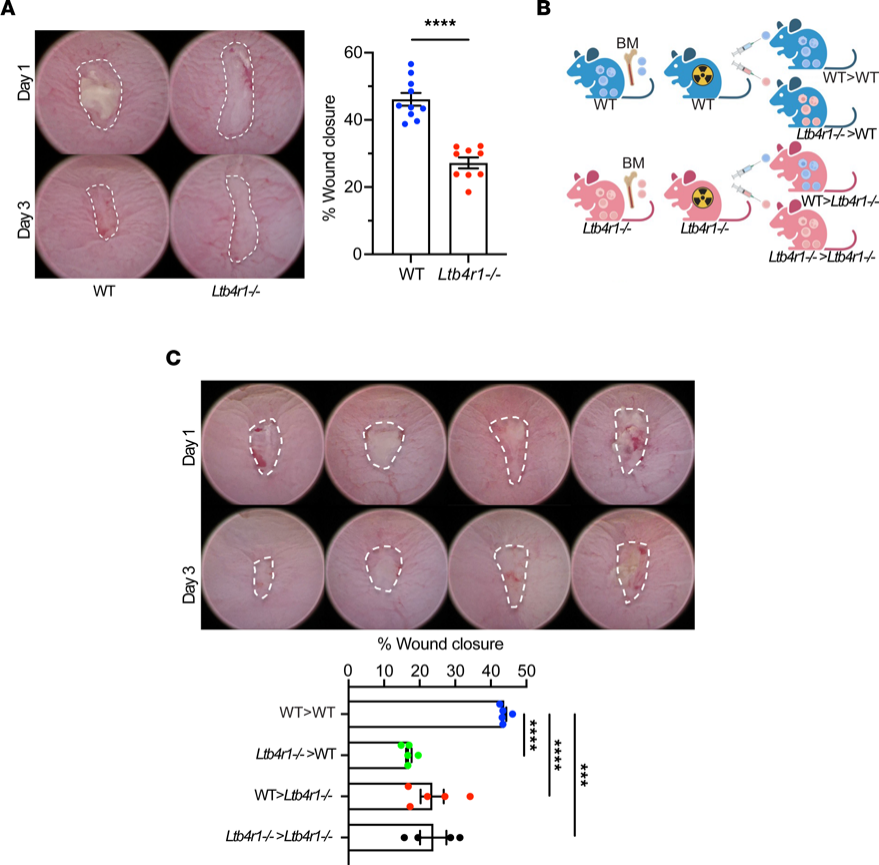
Role of BLT1 in intestinal mucosal wound repair in vivo. (**A**) In vivo intestinal mucosal wound repair in *Ltb4r1^–/–^* mice. Utilizing a miniature video endoscope and biopsy scissors, 5 wounds were created in the dorsal aspect of the colonic mucosa of anesthetized mice. Digital images of wound surface area at 1 and 3 days after wounding are shown (left). Points represent the mean value within all wounds from individual mice (right). The data are presented as the mean ± SEM of 9 to 10 mice. Statistical analysis was performed using an unpaired (2-tailed) *t* test with Welch’s correction. ****P < 0.0001. (**B** and **C**) In vivo intestinal mucosal wound repair in BM chimeric mice. (**B**) Illustration of BM chimera experiment. (**C**) Digital images of wound surface area at 1 and 3 days after wounding are shown (left). Points represent the mean value within all wounds from individual mice (right). The data are presented as the mean ± SEM of 5 mice. Statistical analysis was performed using 1-way ANOVA followed by post hoc Welch’s *t* test with Bonferroni’s correction. ***P < 0.001, ****P < 0.0001.

To determine the role of BLT1 in intestinal mucosal wound repair in vivo, we examined intestinal mucosal healing in *Ltb4r1^–/–^* and WT mice using a well-characterized colonic biopsy-induced injury model. As shown in Figure 5A, colonic mucosal wound repair was dramatically delayed in *Ltb4r1^–/–^* mice compared with WT mice 3 days postinjury (46.1% ± 1.9% in WT mice, 27.2% ± 1.6% in *Ltb4r1^–/–^* mice; *P* < 0.0001). The digitally quantified wound healing data were consistent with histological analyses of healing wounds supporting markedly delayed wound closure 3 days after injury in *Ltb4r1^–/–^* mice. Since IECs and immune cells express BLT1 (Figure 1A), we evaluated the relative contribution of these cell types in regulating mucosal wound repair. Irradiated WT or *Ltb4r1^–/–^* recipient mice were reconstituted with BM cells from either donor WT or *Ltb4r1^–/–^* mice to generate chimeric mice (Figure 5B), followed by biopsy-induced mucosal wound repair experiments. As expected, wound closure 3 days postinjury was significantly delayed in WT mice reconstituted with *Ltb4r1^–/–^* BM (*Ltb4r1^–/–^* > WT), supporting that hematopoietic derived (immune) cell–expressed BLT1 plays a role in regulating colonic mucosal wound repair. However, and importantly, *Ltb4r1^–/–^* mice reconstituted with WT BM (WT > *Ltb4r1^–/–^*) also had a similar delay in wound healing responses (Figure 5C), which is consistent with an equivalent nonhematopoietic (e.g., epithelial) derived BLT1 response in regulating intestinal mucosal wound repair.

## Discussion

Active and coordinated repair responses that promote migration and proliferation of IECs are essential to cover denuded mucosal surfaces and reestablish intestinal mucosal barrier function. These re-epithelization events are facilitated by interactions between mediators derived from epithelium and immune cells in the injured intestinal mucosa and their receptors (21). This study identifies expression of the RvE1/LTB_4_ receptor BLT1 in the intestinal epithelium and demonstrates a critical role of IEC-expressed BLT1 and LTB_4_ in regulating epithelial wound repair.

It is well appreciated that another important ligand for BLT1 and CMKLR1 is RvE1. In this report, using in vivo RNAscope in situ hybridization, we observed that IECs preferentially expressed *Ltb4r1* (BLT1 gene) and not *Cmklr1* mRNA, whereas lamina propria cells expressed mRNA for both these receptors. Human and murine colonic epithelia displayed robust expression of *BLT1* mRNA at the base of the crypt under normal conditions, and expression was highly upregulated after mucosal injury in response to the inflammatory milieu in the wound bed. BLT2 but not BLT1 expression by IECs has been reported with only a few reports showing BLT1 expression by IECs mostly related to carcinoma progression (18). Our expression and pharmacological in vitro studies suggest that ligation of BLT1 and not CMKLR1 by RvE1 mediates intestinal epithelial prorepair effects, suggesting that BLT1 in IECs acts as an active receptor for RvE1. While we previously reported increased expression of *Cmklr1* mRNA in murine repairing colonic mucosal wounds (4) that supports a role of CMKLR1 in mucosal wound repair, these findings are consistent with CMKLR1 playing an important role in immune cell signaling that contributes to intestinal mucosal wound healing.

Spatiotemporal analysis identified expression of *LTB4R*/*Ltb4r1* in both human and murine IECs, supporting a ubiquitous localization of BLT1 on cell types not previously reported to our knowledge. We observed an enrichment of *Ltb4r1* mRNA expression in IECs located at the base of the crypts in murine colonic tissue that was upregulated after biopsy-induced mechanical injury of the mucosa. Using primary human and murine colonoids, and analogous to tissue labeling experiments where *BLT1* mRNA was identified in proliferating epithelial cells at crypt bases, we observed increased expression of *LTB4R*/*Ltb4r1* in proliferative colonoids grown in 3D structures compared with differentiated colonoids that recapitulate luminal epithelial cells.

We have previously reported that the proinflammatory cytokine TNF-α increases intestinal epithelial wound repair that is in part mediated by the cytokine-induced upregulation of prorepair GPCRs (19). We observed that TNF-α, in combination with IFN-γ, stimulated increased expression of *LTB4R* in human IECs. Importantly, these cytokines also enhanced prorepair effects of epithelial BLT1, suggesting “proinflammatory” mediators, such as LTB_4_ and TNF-α, have very important “prorepair” properties in IECs. Our findings strongly support the current concept that inflammation not only is important for host defense but also plays a pivotal role in setting the stage for tissue repair. Our studies support a paradigm shift where proinflammatory mediators often seen as damaging molecules play a pivotal role in initiation tissue repair. Controlled inflammation is clearly essential for host defense. Proinflammatory mediators, often perceived as damaging and detrimental, set the stage for resolution of inflammation and facilitating reparative events required for restoring tissue homeostasis. These highly regulated mechanisms are perturbed in chronic inflammatory diseases that are associated with impaired tissue repair. Thus, an improved understanding of how proinflammatory soluble mediators create the bridge to repair will help in the rational design of therapies to promote wound healing.

Mucosal tissues obtained from people with chronic IBD have increased expression of both LTB_4_ and BLT1 (16–18). We observed that epithelial *LTB4R* expression was higher in the crypts from individuals with IBD compared with healthy controls. The LTB_4_/BLT1 axis may thus play a role in the impaired wound repair responses observed in chronically inflamed mucosa as seen in IBD. Here we demonstrate that IEC-expressed BLT1 has a beneficial role in promoting acute colonic wound repair, but more work is needed to understand the role of BLT1 in chronic intestinal inflammation–induced injury.

Given the marked upregulation of BLT1 in healing wounds, we analyzed the specific contribution of BLT1 to IEC repair. Mucosal wound repair requires coordinated migration of epithelial cells from crypts adjoining wounds. During repair, epithelial cells undergo morphologic changes in shape, modify cell-cell contacts, and migrate collectively to reseal the barrier (1). Given the importance of polarized epithelial cell migration to achieve wound repair, we analyzed the influence of LTB_4_ on directional migration of epithelial cells using DiPer software (20) (22, 23). These analyses suggest that LTB_4_ signaling regulates collective IEC migration through enhanced directional persistence and speed of cell movement. It is also well appreciated that remodeling of the actin cytoskeleton and integrin-containing focal cell matrix adhesions plays a pivotal role in controlling forward movement of cells (21). Our studies revealed that LTB_4_-mediated ligation of BLT1 activates proteins that control remodeling of focal adhesions. Furthermore, we observed that LTB_4_ exposure enhanced proliferation of colonoids that likely contribute to observed prorepair properties of LTB_4_/BLT1 signaling. Other studies have reported that BLT1 signaling enhances proliferation of other cell types, including B cells (24), hepatocytes (25), and smooth muscle cells (26). These reports support our findings that an LTB_4_/BLT1 signaling axis likely promotes proliferation of IECs. Importantly, we observed delayed wound healing in 2D colonoids deficient in BLT1, suggesting that epithelial cells may produce LTB_4_ in an autocrine fashion to promote wound healing or that epithelial BLT1 directly regulates expression of molecules involved in the repair process. Proinflammatory leukotrienes, generated by 5-lipoxygenase (5-LOX) and the 5-LOX–activating protein, initiate and maintain inflammation while SPMs generated by various LOXs promote resolution and repair (27, 28). Since 5-LOX also contributes to SPM biosynthesis, pharmacological manipulation of the 5-LOX pathway and activation of 12-/15-LOXs might cause suppression of leukotriene formation and maintain SPM generation. Previous reports suggested that 5-LOX inhibitors increase wound healing by decreasing LTB_4_ synthesis and neutrophil recruitment (29, 30). Interestingly, we and others have shown that neutrophil depletion during acute injury causes delayed repair, implying that 5-LO inhibition in early stages of colonic wound healing is detrimental for mucosal repair (31). 5-LO–KO mice exhibit faster skin wound healing compared with WT mice (32). However, pharmacological inhibition of 5-LO in vitro inhibits migration and proliferation of keratinocytes (29), suggesting that the role of 5-LO during epithelial wound repair is complex and might depend on the tissue-specific molecular interactions in the wound milieu. Furthermore, 5-LO also regulates the synthesis of antiinflammatory soluble mediators such as SPMs, and therefore inhibiting these molecules would also impact reparative responses.

Finally, we observed that BLT1 signaling plays an important role in regulating in vivo intestinal mucosal wound repair. BM transplant experiments and analyses of colonic mucosal wound repair results identified similar contributions of both IEC and immune cell–expressed BLT1 in regulating intestinal mucosal wound repair. It is important to note that neutrophils are the first responders to sites of acute injury in the mucosa (1). In support of this, we observed abundant neutrophils in murine colonic mucosal wounds within 4 to 6 hours after initial injury, with maximum numbers detected between 6 and 24 hours after biopsy-induced injury (33, 34). Neutrophils play a critical role in facilitating recovery since their depletion results in impaired mucosal repair and delayed recovery from colitis (35, 36). Neutrophils are also major producers of SPMs, and the LTB_4_/BLT1 pathway is well known for its function as a chemotactic signal that regulates neutrophil migration to sites of inflammation (15). Since we previously showed that LTB_4_ levels are increased in acute colonic mucosal wounds compared with intact tissues (4), and infiltrating leukocytes are a potent source of LTB_4_, we therefore suggest that LTB_4_ released in the wound bed engages epithelial BLT1 and triggers intestinal epithelial wound healing. The concentration of LTB4 at sites of mucosal injury is much higher than the other BLT1 ligand RvE1. Interestingly, kinetics of the levels of LTB_4_ and RvE1 in wounds are also different. LTB_4_ is secreted during the early stages of inflammation while RvE1 is released at later points when LTB_4_ synthesis is declining. As mentioned above, inflammation and repair are complementary events that are initiated at the same time to orchestrate repair. While LTB_4_ promotes migration of immune cells to sites of mucosal injury, it also enhances migration of epithelial cells. Neutrophils express both BLT1 and CMKLR1, while IECs express only BLT1. RvE1 signaling through CMKLR1 and BLT1 promotes PMN apoptosis, and in IECs RvE1 sustains the migratory response triggered by BLT1 activation by LTB_4_. Our finding that LTB_4_ can signal on epithelial cells and trigger prorepair responses challenges the long-standing dogma that LTB_4_/BLT1 signaling is exclusively a proinflammatory event. Taken together, our findings highlight a potentially novel intestinal epithelial prorepair mechanism that is mediated by the LTB_4_/BLT1 signaling pathway, which serves to orchestrate mucosal wound repair and restore the critical mucosal barrier.

## Methods

### Mice.

*Ltb4r1^–/–^* mice (B6.129Sa-*Ltb4r1tm1Adl*/J) on a C57BL/6 background (11) were purchased from The Jackson Laboratory. All mice were housed in the experimental animal facility at the University of Michigan and were provided free access to food and water. All experiments were performed in accordance with the *Guide for the Care and Use of Laboratory Animals* of the NIH (National Academies Press, 2011) and the University of Michigan.

### Colonic organoid and epithelial monolayer culture.

Human 3D colonic organoids (colonoids) were provided from Translational Tissue Modeling Laboratory (University of Michigan) and maintained in the laboratory (37). Murine colonoids were created and maintained in culture according to our previous report (38) with modified methods reported by Sato et al. (39). Isolated intestinal crypts from WT or *Ltb4r1^–/–^* mice were embedded in Matrigel and maintained in LWRN complete media (40). 2D colonic epithelial monolayers from human or murine 3D colonoids were generated as previously described (40) and maintained in LWRN complete media.

### Cell lines.

The human IECs, SKCO-15 and T84, were cultured as described previously (19). In some experiments, SKCO-15 cells were stimulated with 100 ng/mL IFN-γ (catalog 285-IF, R&D Systems) and 100 ng/mL TNF-α (catalog 210-TA, R&D Systems).

### RNAscope in situ hybridization.

RNAscope was performed on frozen tissue sections of human and murine colonic mucosa. In situ hybridization was performed according to the protocol of the RNAscope Multiplex Fluorescent Reagent Kit v2 (catalog 323100, Advanced Cell Diagnostics). In this study, positive (*Homo sapiens* *PPIB* or *Mus musculus* *Ppib*) and negative (*Bacillus subtilis* strain SMY *DapB*) control probe and 4 different probes (human *LTB4R* and *CMKLR1* and mouse *Ltb4r1* and *Cmklr1*) were used. Images were acquired using a Nikon A1 confocal microscope (Nikon). Quantification of *Ltb4r1* in the murine colonic mucosa was analyzed using QuPath (v0.3.0) as recommended by Advanced Cell Diagnostics.

### RNA extraction and qPCR.

The mRNA expression levels of various genes were measured in human and mouse samples as described previously (41). In brief, total RNA was extracted from the samples using the RNeasy Mini Kit (catalog 74106, QIAGEN) according to the manufacturer’s instructions. Reverse transcription was performed using the iScript Reverse Transcription Supermix for RT-qPCR (catalog 1708840, BioRad). qPCR amplification was then performed using the iQ SYBR Green Supermix (catalog 1708880, Bio-Rad) in a CFX Connect Real-Time PCR Detection System (Bio-Rad). Target mRNA levels were normalized to those of *TBP* or *Tbp* as the internal control in each sample and calculated by the 2^-ΔΔCt^ method. The results are expressed as ratios relative to the average for the control group. The following primer pairs were used: *Homo sapiens* LTB4R, (forward) 5′-GTTTTGGACTGGCTGGTTGC-3′ and (reverse) 5′-GGTACGCGAGGACGGGTGTG-3′; *Homo sapiens* CMKLR1 (ACAGCATCACTTCTACCACTT) 5′–3′ and (GAGTCCTCAGCCAATCAGTC) 5′–3′; *Homo sapiens* TBP, (forward) 5′-TGCACAGGAGCCAAGAGTGAA-3′ and (reverse) 5′-CACATCACAGCTCCCCACCA-3′; *Mus musculus* Ltb4r1, (forward) 5′-ATGGCTGCAAACACTACATCTC-3′ and (reverse) 5′-GACCGTGCGTTTCTGCATC-3′; *Mus musculus* Tbp, (forward) 5′-GGAATTGTACCGCAGCTTCAAA-3′ and (reverse) 5′-GATGACTGCAGCAAATCGCTT-3′ (Integrated DNA Technologies).

### Wound healing assay.

For in vitro experiments, SKCO-15, and primary human and murine colonoids cultured as 2D monolayers, were subjected to scratch wounding assays. Monolayers were cultured on 48-well tissue culture plates (Corning) to confluence and scratched using a 10 μL pipette tip. In the case of colonoids, monolayers were cultured on collagen-coated (catalog C5533, MilliporeSigma) 48-well tissue culture plates. Medium was changed after wounding and video quantification of scratch wound closure was performed by imaging wounds at 1-hour intervals in Axio Observer Z1 live-cell microscopy system (ZEISS). IECs were incubated with LTB4 (Cayman Chemical) or RvE1 (Cayman Chemical) for 24 hours. BLT1 antagonist (CP105,696; MilliporeSigma) or CMKLR1 antagonist (α-NETA; Cayman Chemical) was applied 30 minutes before LTB4 or RvE1 treatment. Wound closure was quantified at the indicated time points using ImageJ software (NIH). For in vivo wounding experiments of colonic mucosa, a biopsy-based mucosal wound model was employed using a high-resolution, miniaturized endoscope system (Coloview Veterinary Endoscope; Karl Storz) equipped with biopsy forceps to create biopsy-induced injury of the colonic mucosa at 5 sites along the dorsal aspect of the colon of anesthetized mice (i.p. injection of 100 mg/kg ketamine and 5 mg/kg xylazine). Wound healing was quantified at 1 day and 3 days after injury. Endoscopic procedures were viewed with high-resolution (1,027 × 768 pixels) images on a flat-panel color monitor. Each wound region was digitally photographed at 1 day and 3 days, and wound areas were measured using ImageJ software.

### Epithelial cell migration assay (DiPer).

For time-lapse experiments, cells were imaged for 12 hours at a time every 30 minutes. Images were exported and stacked to videos. Cellular tracking was performed using 20 cells from each sample (10 cells/each side) using ImageJ software. Data were analyzed via DiPer for Plot_At_Origin (plots cell trajectories emanating from the origin), MSD, direction autocorrelation, and cell speed (20).

### Immunoblot.

For cell lysis, IEC monolayers were harvested in RIPA buffer as described previously (4). The following antibodies were used: FAK (catalog 610088) BD Biosciences; p-FAK (Y861) (catalog PS 1008) Calbiochem; p-FAK (Y397) (catalog 3283) and p-FAK (Y925) (catalog 3284); and Src (catalog 2108) and p-Src (y416) (catalog 2101) Cell Signaling Technology.

### Epithelial cell proliferation assay.

Two hours before fixing of cells, EdU was added to the media at a concentration of 100 μM. Proliferating cells were detected with the Click-iT EdU Cell Proliferation Kit for Imaging and Alexa Fluor 488 dye (catalog C10337, Thermo Fisher Scientific) according to the manufacturer’s instructions and captured using a Nikon A1 confocal microscope.

### BM transplantation.

For total BM transplant experiments, donor BM cells were harvested from WT and *Ltb4r1^–/–^* mice. Recipient mice were sublethally irradiated using 2 times 5 Gy x-rays 4 hours apart (42). A total of 1 × 10^6^ donor BM cells were transplanted by retro-orbital venous plexus injection into recipient mice. Blood samples were collected from the recipients 8 weeks after BM transplantation to confirm engraftment. Experiments using the recipients were conducted 8 weeks after BM transplantation, and blood samples were collected for engraftment and complete blood cell analysis.

### Docking simulation.

For docking studies, BIIL260 was removed from the crystal structure of Protein Data Bank ID: 5x33 (43) to create apo-BLT1 structure, and we predicted the binding site of RvE1 to BLT1 using AutoDock Vina (Scripps Research).

### Statistics.

The data are presented as the mean ± SEM. Statistical analyses were performed with Prism 9 (GraphPad Software) using 1- or 2-way ANOVA followed by Bonferroni’s multiple-comparison test, Tukey’s multiple-comparison test, or an unpaired (2-tailed) *t* test with Welch’s correction. Values of *P* < 0.05 were considered to indicate significant differences.

### Study approval.

All experimental procedures involving animals were conducted in accordance with NIH guidelines and protocols approved by the University Committee on Use and Care of Animals at the University of Michigan.

## Author contributions

SH and MQ performed experiments in addition to data analysis/interpretation. SH and MQ wrote the manuscript. CKM provided technical support and provided human primary cell cultures. ST and SPH helped designed and perform the bone marrow transplant experiments. MO performed in silico docking analysis for BLT1 ligands. MQ, CAP, and AN oversaw the project design and execution, edited the manuscript, and acquired funding.

## Supplementary Material

Supplemental data

## Version 1 10/27/2022

In-Press Preview

## Version 2 12/08/2022

Electronic publication
