# Supplementary material for: Intestinal epithelial BLT1 promotes mucosal repair
Source: JCI Insight. 2022 Dec 8;7(23):e162392. doi: 10.1172/jci.insight.162392 (PMC9746898; doi:10.1172/jci.insight.162392)
Supplement: Supplemental data [file jciinsight-7-162392-s050.pdf]

Fig. S1. BLT1 functions as an epithelial receptor for RvE1

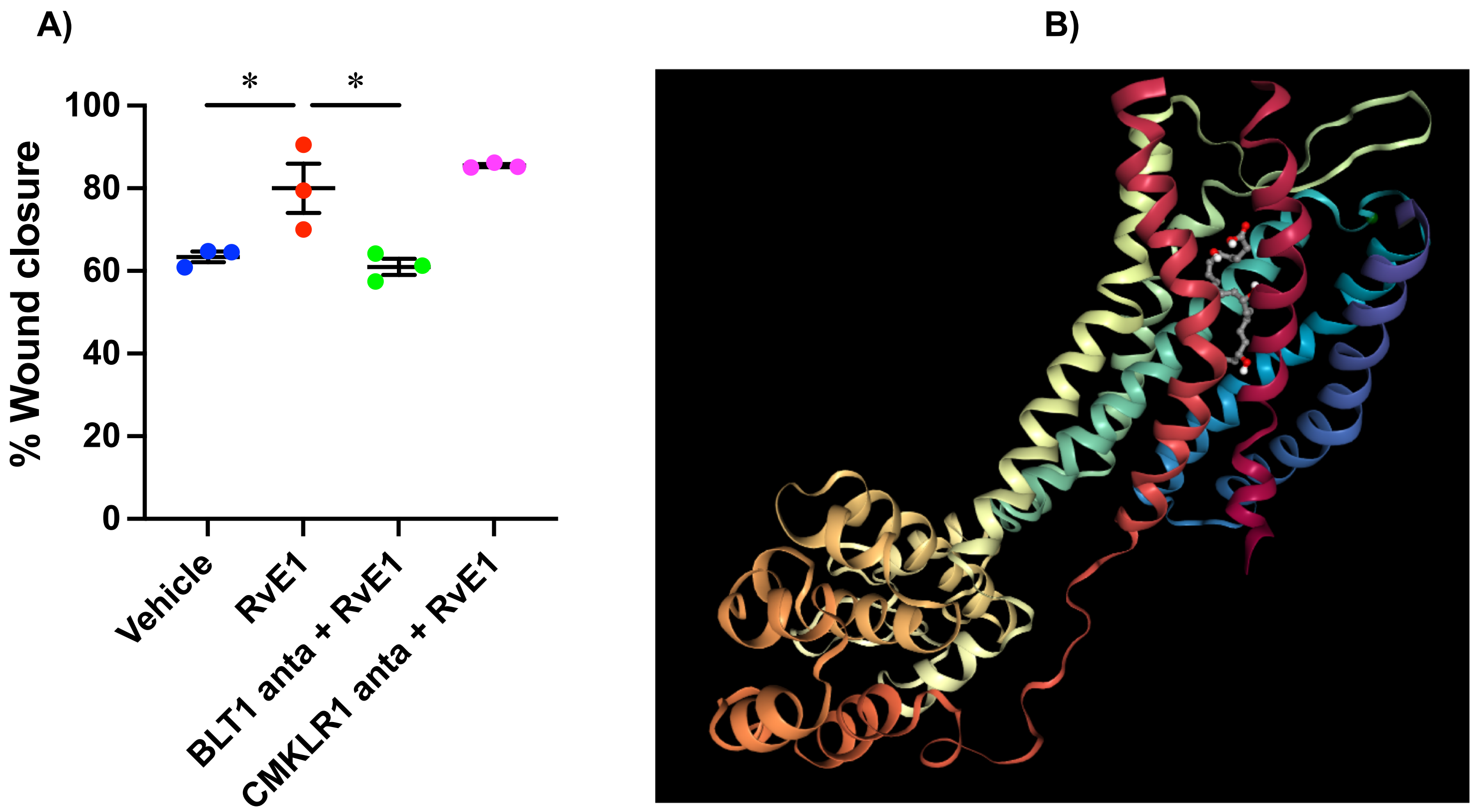

Figure S1. BLT1 functions as a major epithelial receptor for RvE1. (A) Effect of BLT1 or CMKLR1 antagonist on the pro-repair activity of RvE1 in the scratch wound assay using murine primary IECs. After scratch wound was produced, IECs were incubated with RvE1 (100 nM) for 24h. BLT1 (1 μM) or CMKLR1 (10 μM) antagonist was applied 30 min before RvE1 treatment. Quantification of wound repair at 24 h after wounding is shown. The data are presented as the mean ± SEM. Statistical analysis was performed using one-way ANOVA followed by post-hoc Welch's t test with Bonferroni's correction. *$P < 0.05$. (B) Estimated conformation of the protein-ligand complexes for BLT1 and RvE1 calculated by AutoDock Vina.

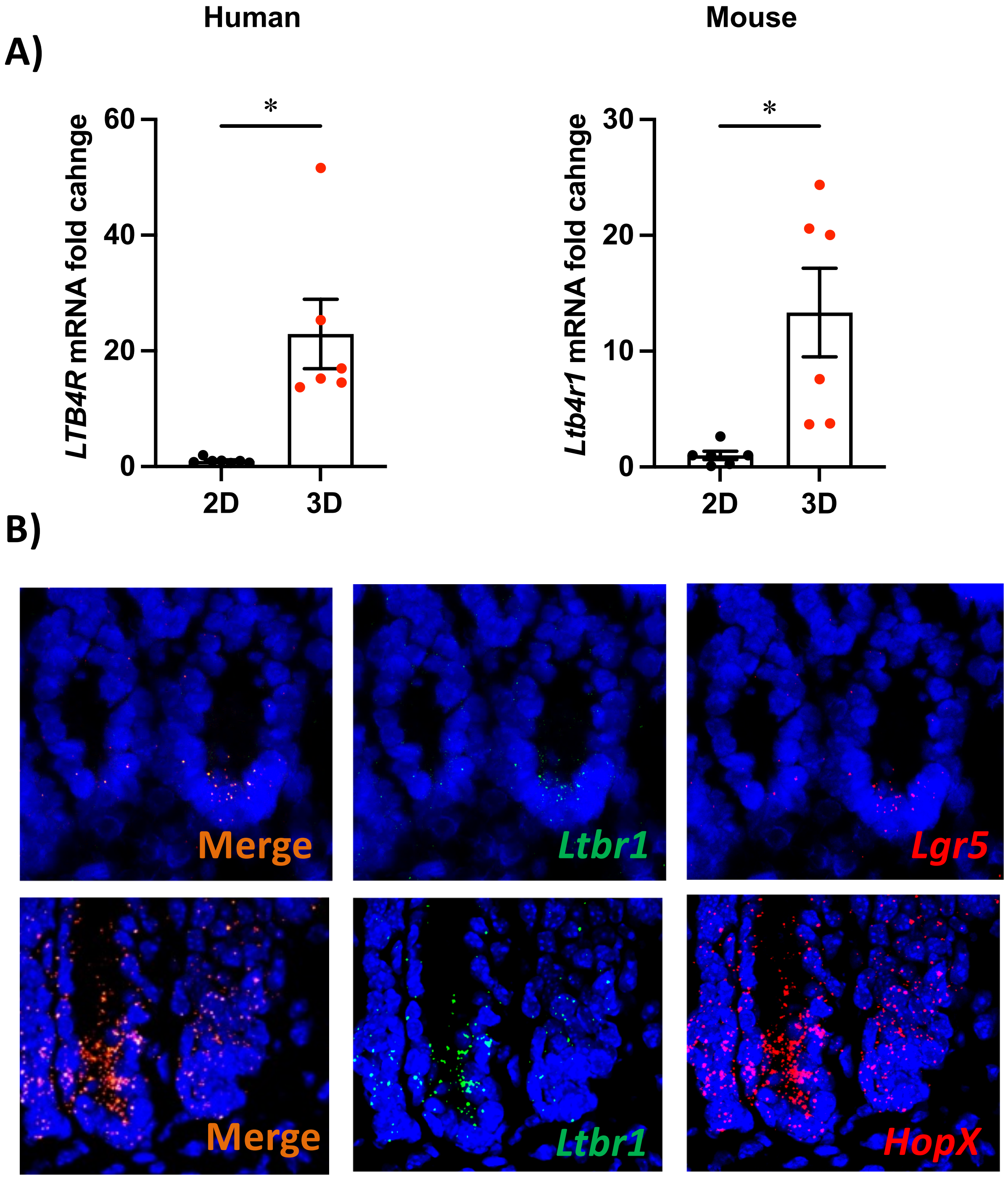

Figure S2. 3D colonoids have a higher expression of mRNA of BLT1 than 2D colonoids. (A) qPCR analysis of BLT1 mRNA level in 2D and 3D cultured colonoids of human or mice. The data are presented as the mean ± SEM. Statistical analysis was performed using an unpaired (two-tailed) t test with Welch's correction. $^*P < 0.05$. B) Intestinal Epithelial BLT1 is expressed at the stem-cell compartment in the colonic crypt base during homeostasis

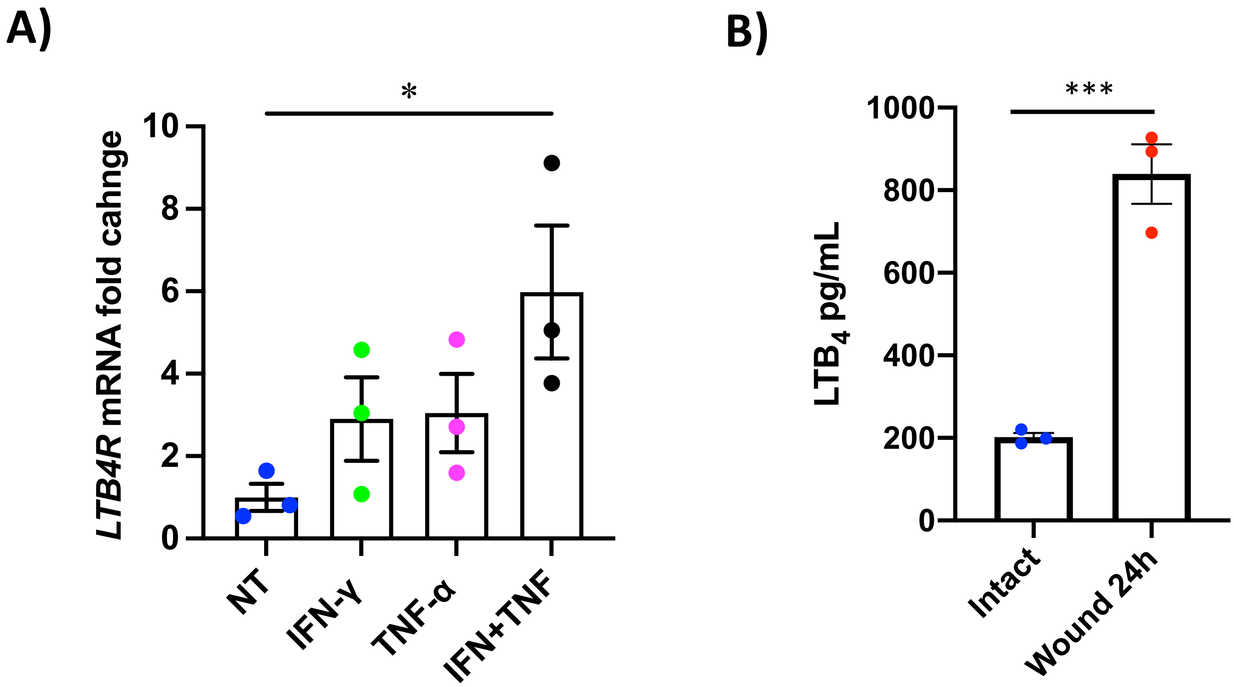

Figure S3. Inflammatory stimulation increases *LTB4R* mRNA expression in human 3D colonoids and increases in-vivo LTB_4 secretion. (A) qPCR analysis of the changes in the expression of *LTB4R* mRNA in the human 3D cultured colonoid stimulated with IFN-γ (10 ng/ml) and/or TNF-α (10 ng/ml) for 24h. The data are presented as the mean ± SEM. Statistical analysis was performed using one-way ANOVA followed by post-hoc Welch's t test with Tukey's correction. *P < 0.05. (B) A colonic mucosal injury causes a dramatic increase in LTB_4 secretion at the wound bed. Statistical analysis was performed using an unpaired (two-tailed) t test with Welch's correction. ****P* < 0.001

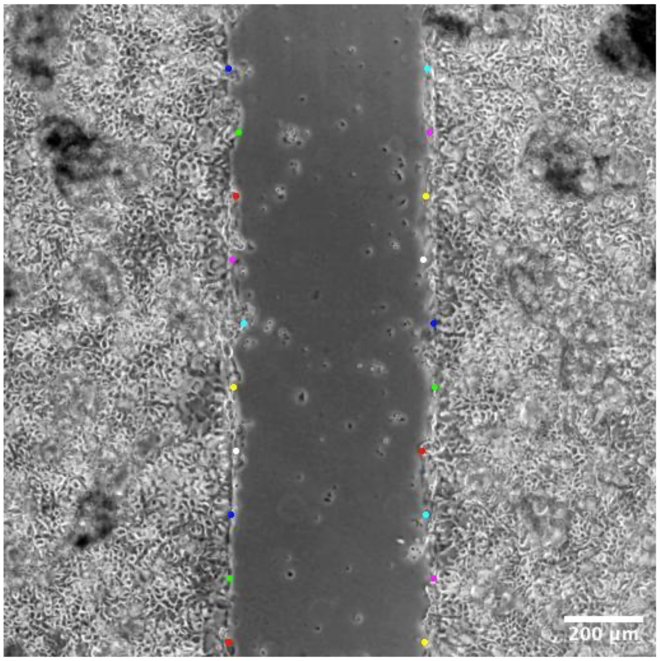

WT (Vehicle)

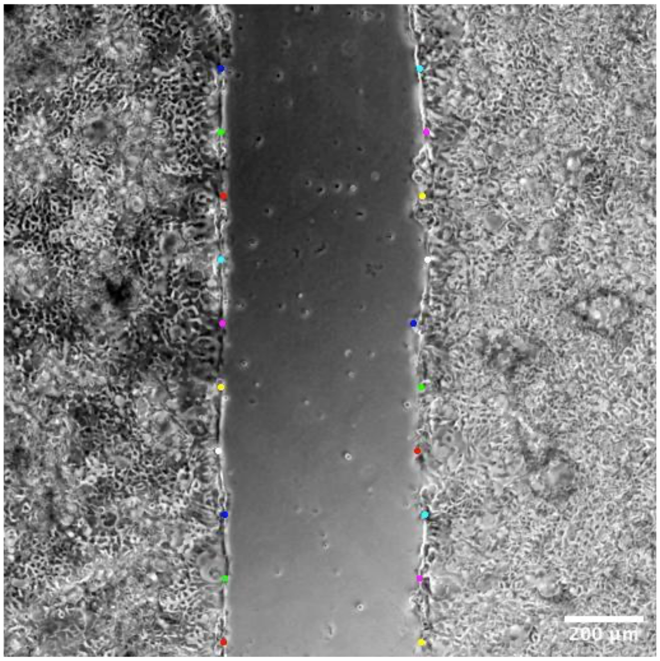

WT (LTB_4)

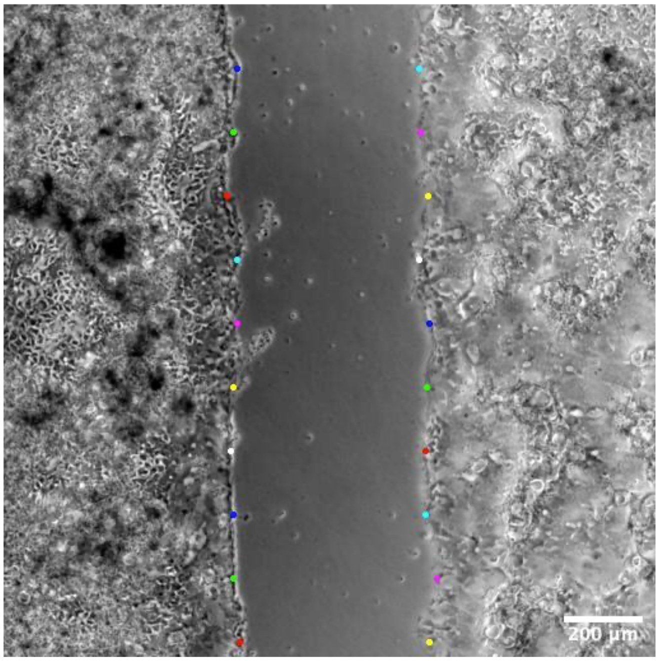

***Ltb4r1-/-* (Vehicle)**

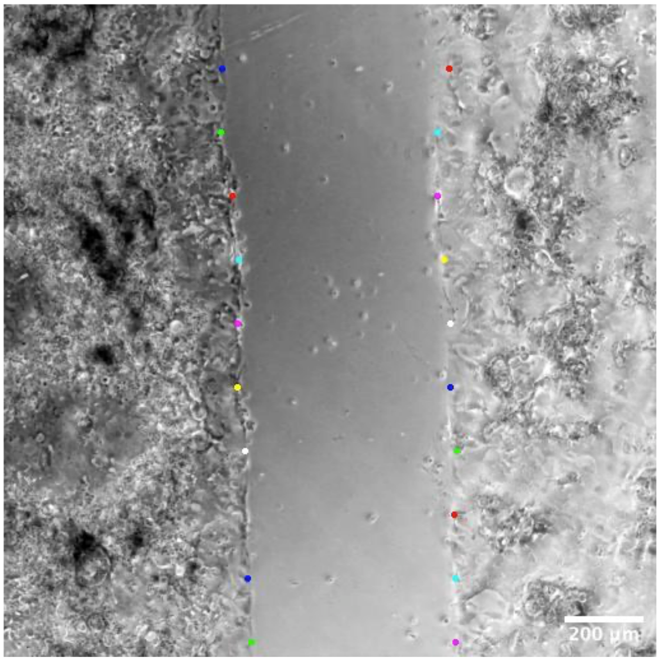

***Ltb4r1-/-* (LTB_4)**

Figure S4. BLT1 activation promotes migration of IECs. Representative tracking movies of IEC movement after wounding are shown (see video). Images were taken 30 minutes apart for video microscopy. Scale bar is 200 µm.

Fig. S5. BLT1 activation promotes proliferation of IECs

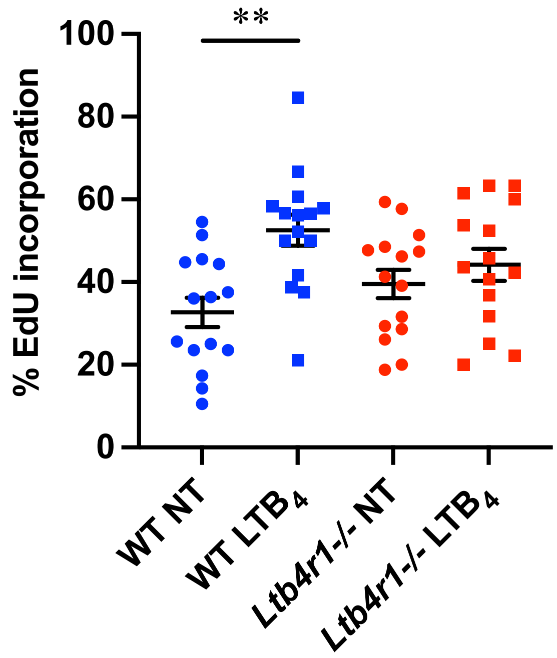

Figure S5. BLT1 activation promotes proliferation of IECs. EdU incorporation analysis in the murine 3D cultured colonoids stimulated with LTB4 (10 nM) for 24h. Comparison of WT and Ltb4r1-/- mice. The data are presented as the mean ± SEM. Statistical analysis was performed using one-way ANOVA followed by post-hoc Welch's t test with Bonferroni's correction. **$P < 0.01$.

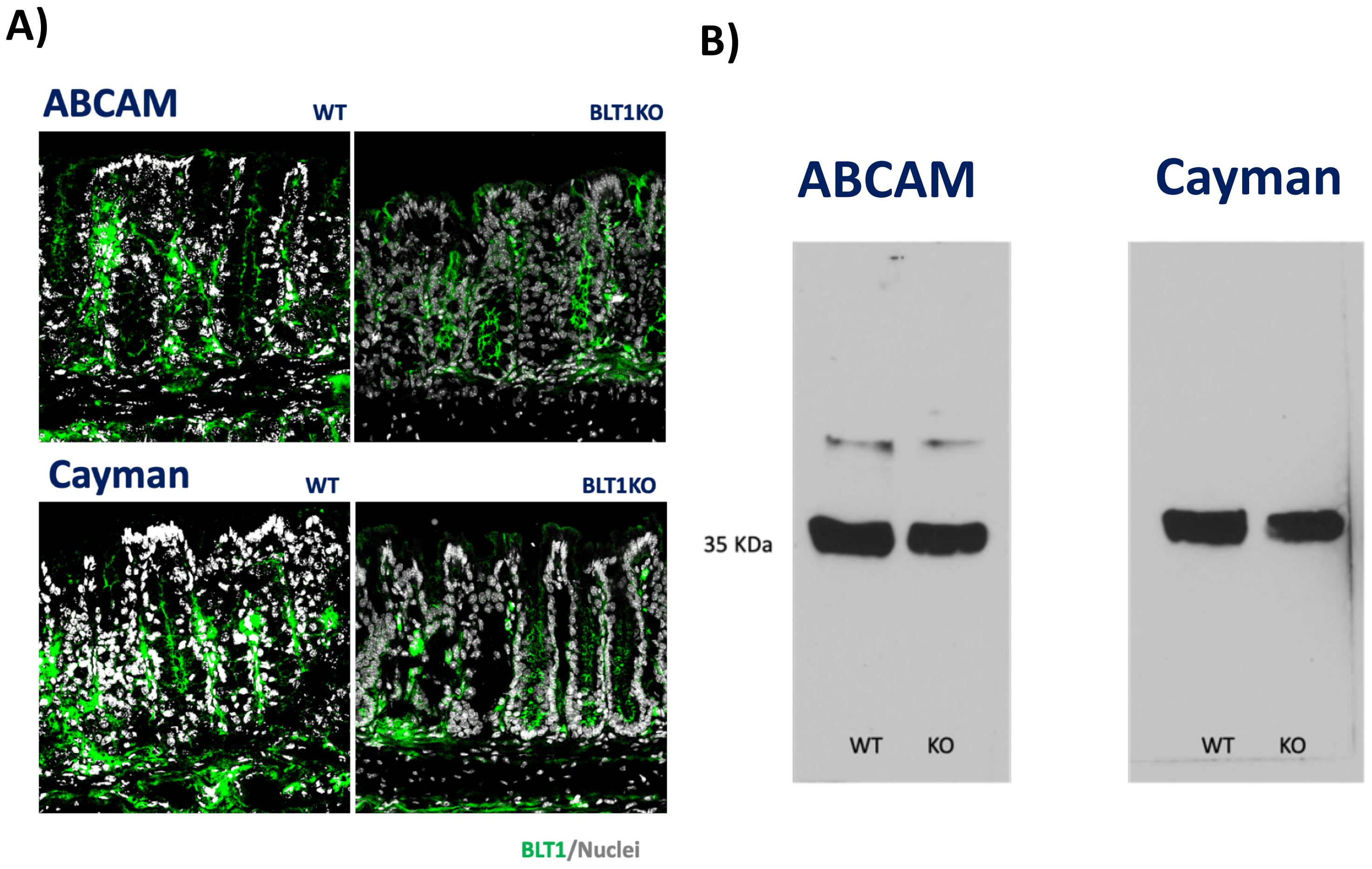

Fig. S6. BLT1 commercially available antibodies are unspecific. Commercially available antibodies shown to be unspecific towards BLT1 by immunofluorescence (A) and western blot (B)
